# Value of the Overall Pneumococcal Polysaccharide Response in the Diagnosis of Primary Humoral Immunodeficiencies

**DOI:** 10.3389/fimmu.2017.01862

**Published:** 2017-12-20

**Authors:** Benjamin Lopez, Mathilde Bahuaud, Claire Fieschi, Souad Mehlal, Mohamed Jeljeli, Stéphanie Rogeau, Séverine Brabant, Anne-Sophie Deleplancque, Sylvain Dubucquoi, Sandrine Poizot, Louis Terriou, David Launay, Frédéric Batteux, Myriam Labalette, Guillaume Lefèvre

**Affiliations:** ^1^CHU Lille, Institut d’Immunologie, Lille, France; ^2^Univ. Lille, U995 – LIRIC – Lille Inflammation Research International Center, Lille, France; ^3^CHU Hôpital Cochin, Laboratoire d’Immunologie Biologique, Plateforme d’Immuno-monitoring Vaccinal, AP-HP, Paris, France; ^4^Sorbonne Paris Cité, Université Paris Diderot, Hôpital Saint-Louis, Service d’Immunopathologie Clinique, Paris, France; ^5^CHU Lille, Département de Médecine Interne et Immunologie Clinique, Centre National de Référence Maladies Systémiques et Auto-immunes Rares, Lille, France

**Keywords:** primary immunodeficiency, humoral immunodeficiency, pneumococcal polysaccharide response, serotype-specific assay, polysaccharide response, overall assay

## Abstract

**Background:**

An overall response assay [OVA, based on a 23-valent pneumococcal polysaccharide vaccine (PPV23)] is widely used to screen for anti-pneumococcal antibodies. Given the heterogeneity of response from one polysaccharide (PS) to another, a World Health Organization-standardized serotype-specific enzyme-linked immunosorbent assay (SSA) is considered to be the only reliable method for testing anti-PS antibody responses in individuals with suspected primary immunodeficiencies (PIDs).

**Objective:**

To evaluate the OVA relative to the reference SSA.

**Methods:**

Serum samples of adult patients referred for a suspected PID were collected before and then 4–8 weeks after immunization with PPV23. The anti-pneumococcal response was systematically assessed with an SSA (7–16 serotypes) and interpreted according to the American Academy of Asthma, Allergy and Immunology’s current guidelines. We used receiver operating characteristic curves and agreement indices to assess the OVA’s diagnostic value in a first cohort. In order to validate these findings, a second (validation) cohort was then prospectively included.

**Results:**

Sixty-two adult patients were included, and 42 (67.7%) were defined as poor responders according to the SSA. Only the post-immunization titer in the OVA was able to correctly identify poor responders; a titer below 110 mg/L gave a positive predictive value of 100% [identifying 24 (57.1%) of the 42 poor responders], and similar levels of diagnostic performance were observed in the validation cohort. The pre-vaccination antibody titer, the post/pre-vaccination antibody titer ratio and a post-vaccination titer above 110 mg/L in the OVA were not predictive of the response in the SSA.

**Conclusion:**

A post-vaccination antibody titer below 110 mg/L in the OVA was constantly associated with a poor PPV23 response using the SSA. In all other cases, SSA is the only reliable method for assessing diagnostic vaccination with PPV23.

## Introduction

An altered immunoglobulin G (IgG) antibody response to vaccines is an important criterion in the diagnosis of primary immunodeficiencies (PIDs), such as common variable immunodeficiency (CVID), transient hypogammaglobulinemia of infancy, selective IgA deficiency, IgG subclass deficiencies, or selective anti-polysaccharide antibody deficiency (SPAD) (1, 2). Although responses to protein or protein-conjugated antigens may be conserved, responses to polysaccharides (PSs) are usually impaired. This is particularly true for SPAD, which is characterized by an isolated defect in the specific anti-polysaccharide IgG response, normal total IgG, IgA/M, and IgG subclass titers, and the absence of a T-cell deficiency (1, 2). The response to PS antigens is usually tested by challenging the patient with the pneumococcal capsular polysaccharides (PnPSs) in the 23-valent pneumococcal vaccine (PPV23); in 2012, the American Academy of Asthma, Allergy and Immunology (AAAAI) proposed consensus criteria for an impaired response to PnPSs (1, 2). The currently accepted gold-standard test for specific anti-PnPS IgG responses is the serotype-specific enzyme-linked immunosorbent assay (ELISA) validated by the World Health Organization (WHO) (3). Given that each serotype requires a single ELISA, the serotype-specific assay (SSA) is too expensive and time-consuming to be used in routine practice; as a result, it has not been widely disseminated and is available in only a few highly qualified laboratories. Several alternative in-house multiplexed approaches have also been suggested but they are of limited access (4–7).

By contrast, the overall anti-PnPS response assay (OVA, which measures the antibody response to all 23 of the PPV23’s antigens) is widely available in hospital central laboratories and private diagnostic laboratories as in-house assays or easy-to-use commercial kits (8). In view of its design, the OVA only characterizes the overall response to all serotypes and does not take account of the well-known inter-serotype heterogeneity in antibody titers (9, 10). The published normative values were calculated for healthy, non-vaccinated control groups. Although several pathological values have also been reported, they were calculated from groups of patients with greatly differing diagnostic criteria (8, 11–13). The clinical relevance of OVA with regard to SSA has not previously been assessed, and OVA is not currently recommended for the assessment of anti-PnPS responses.

In this context, the present study was designed to assess the value of the OVA (the index test) in the diagnosis of a defective PS vaccine response in adults, relative to the gold-standard SSA.

## Materials and Methods

### Study Design

We first performed a retrospective study in patients being tested with the WHO-validated reference SSA for the diagnosis of suspected PIDs. In order to validate the threshold determined in the retrospective cohort, a second, separate cohort of patients with suspected PID was then prospectively evaluated.

### Participants

In the retrospective study, the population was composed of adult patients having undergone a PS vaccine response assay for a suspected PID in the Department of Clinical Immunology at Lille University Medical Center (Lille, France), Saint Louis University Medical Center (Paris, France) and/or patients included in an ongoing trial which aims to evaluate a screening strategy of PIDs in adults (ClinicalTrials ref: NCT02972281).

In the prospective validation study, a second cohort of consecutive adult patients referred for possible PID from the above-mentioned ongoing NCT02972281 trial was included on a blind basis.

In both studies, stored serum samples were retrieved from the Immunomonitoring Facility at Cochin University Medical Center (Paris, France). The inclusion criteria were unexplained recurrent and/or severe bacterial infections with encapsulated bacteria (i.e., upper and/or lower respiratory tract infections, invasive infections). The main exclusion criteria were as follows: evidence of secondary immunodeficiency, immunoglobulin replacement therapy, non-compliance with the post-immunization period, and pneumococcal immunization in the previous 5 years. All identified PIDs were classified according to current guidelines (1, 14).

### Ethics

Both studies were conducted in accordance with the recommendations of the Helsinki Declaration and complied with the requirements of the French Commission Nationale Informatique et Libertés.

Patients included through the ongoing adult PIDs screening study (ClinicalTrials ref: NCT02972281) gave written informed consent according to the current legislation and the study protocol.

Patients included through routine consultations of French university hospitals received information, and the ability to refuse, that their clinical data and/or remaining biological samples after routine testing completion could be used for research purposes. All samples were retrieved from a human biological collection declared to and authorized by the French Ministry in charge of Research (No. DC-2008-642). Hence, no written informed consent was collected from this population.

### Test methods

#### The Clinical Reference Standard

The PnPS response was tested in the Immunomonitoring Facility at Cochin University Medical Center (Paris, France), using a WHO-validated SSA. Titers of antibody against seven capsular serotypes (4, 6B, 9V, 14, 18C, 19F, and 23F) were assessed before and then 4–8 weeks after the administration of PPV23 (3). The results were interpreted according to the 2012 AAAAI guidelines, which were recently reviewed by a panel of experts (2, 15). Briefly, an insufficient response for a given serotype was considered to correspond to post-immunization antibody titers below 1.3 mg/L or failing to show a fourfold increase. A twofold increase was deemed acceptable if the initial value was already greater than 1.3 mg/L. A poor response to PnPSs was defined by an insufficient response to at least 70% (i.e., 5 or more) of the 7 tested serotypes, and patients were, therefore, classified as “good responders” or “poor responders.” To validate the relevance of the 7-serotype PS response, serum samples with sufficient pre- or post-immunization (PPV23) volumes were tested for antibodies against nine additional serotypes (1, 3, 5, 6A, 7F, 19A, 10A, 12F, and 15B) (data not shown).

#### The Index Test

An OVA was used to measure the total titer of antibodies to all 23 serotypes in the serum samples studied with the reference standard at the time of the initial diagnosis and subsequently stored in a dedicated −80°C serum bank. The assay was performed in the Immunology Institute at Lille University Medical Center (Lille, France), using the VaccZyme™ anti-PCP IgG Enzyme Immunoassay Kit (The Binding Site^®^, Birmingham, United Kingdom) on an ETI–Max 3000™ analyzer (DiaSorin^®^, Vercelli, Italy). The serum samples were analyzed at a first-intention dilution of 1:100. A further 1:10 dilution was performed when antibody titers fell outside the assay’s linearity range. The OVA results were initially interpreted blindly, i.e., without regard to the SSA results. According to the study protocol, indeterminate results and missing data were to be excluded before the analysis.

### Analyses

Analyses were performed using SAS^®^ software (version 9.4; SAS Institute Inc., Cary, NC, USA). Continuous variables are quoted as the median (range) and categorical variables are quoted as the number (percentage). The good responders and poor responders were compared with regard to their clinical and biological characteristics by using a Mann–Whitney test for continuous variables, and the chi-squared test or Fisher’s exact test for categorical variables, as appropriate.

In the retrospective study, linear regression and Pearson’s correlation coefficient were used to assess the putative relationship between OVA antibody titers and the sum of serotype-specific antibody titers before and after vaccination (13, 16). Receiver operating characteristic (ROC) curves were plotted and the area under the curve (AUC) was calculated for pre- and post-immunization antibody titers, the post-immunization increase and the post-/pre-immunization titer ratio. The best cut-off values were then chosen (i) according to the Youden index calculation [which defines the maximum potential effectiveness of a biomarker when equal weight is given to sensitivity (Se) and specificity (Sp)], and (ii) in order to maximize the assay’s Sp value. Positive and negative predictive values were calculated on the basis of the prevalence of the target condition in the included population. For each identified cut-off value, the OVA’s performance and agreement levels were measured using McNemar’s test and Cohen’s kappa.

In the prospective validation study, the best cut-off value determined in the retrospective study was used to classify patients against the gold-standard diagnosis and, thus, validate our methodology. All tests were two-tailed, and the threshold for statistical significance was set to *p* < 0.05.

## Results

### The Retrospective Study

#### Participants

Sixty-two patients were included, 33 (53.2%) were females, and the median (range) age at inclusion was 37.5 (18–75) years. No indeterminate results were observed with either assay. According to the SSA, 42 (67.7%) of the patients showed a poor response to PnPSs. The identified PIDs were CVID (*n* = 5, 8.1%), unclassified isolated IgG deficiency (*n* = 7, 11.3%), IgG subclass deficiency (*n* = 8, 12.9%, including IgG1/3 deficiency, *n* = 3; IgG2 deficiency, *n* = 2; IgG2/IgA deficiency, *n* = 3), isolated IgA deficiency (*n* = 2, 3.2%), isolated IgM deficiency (*n* = 1, 1.6%) and SPAD (*n* = 24, 38.7%). Some of the patients with SPAD have been described elsewhere (17). The patients’ clinical characteristics are presented as a function of the vaccine response in Table 1.

**Table 1:** Clinical and biological characteristics of the retrospective study population as a whole and according to the vaccine response status.

	Whole population	Normal^a^ polysaccharide (PS) response (good responders)	Impaired^a^ PS response (poor responders)	*p*

	*n* = 62	*n* = 20 (32.3%)	*n* = 42 (67.7%)	
**Demographics**				
Age (years)	37.5 (18–75)	30 (18–75)	41 (18–70)	0.065
Gender (women) *n* = (%)	33 (53.2%)	9 (45.0%)	24 (57.1%)	0.370
**Diagnosis *n* = (%)**				
Common variable immunodeficiency	5 (8.1%)	0 (0.0%)	5 (11.9%)	0.165
Unclassified, isolated immunoglobulin G (IgG) deficiency	7 (11.3%)	2 (10.0%)	5 (11.9%)	1.000
IgG sub-class deficiency	8 (12.9%)	2 (10.0%)	6 (14.3%)	1.000
IgG1/3 deficiency	3 (4.8%)	1 (5.0%)	2 (4.8%)	1.000
IgG2 deficiency	2 (3.2%)	1 (5.0%)	1 (2.4%)	0.545
IgG2/A deficiency	3 (4.8%)	0 (0.0%)	3 (7.2%)	0.445
Isolated IgA deficiency	2 (3.2%)	1 (5.0%)	1 (2.4%)	0.480
Isolated IgM deficiency	1 (1.6%)	0 (0.0%)	1 (2.4%)	1.000
Specific PS antibody deficiency	24 (38.7%)	0 (0.0%)	24 (57.1%)	**<0.001**
Noimmunodeficiency diagnosed	15 (24.2%)	15 (75.0%)	0 (0.0%)	**<0.001**
**PS response**				
Time interval between immunization and sample collection (weeks)	6.3 (4.0–7.9)	6.0 (4.0–7.8)	6.4 (4.1–7.9)	0.465
Serotype-specific assay (SSA)				
Number of good responses	3 (0–7)	6 (5–7)	2 (0–4)	**<0.001**
**Overall assay (OVA) (mg/L)**				
Pre-immunization OVA antibody titer	26 (4–196)	47.5 (12–196)	22.5 (4–166)	**0.004**
Post-immunization OVA antibody titer	151 (9–1360)	480 (114–1360)	100 (9–1010)	**<0.001**
Post-immunization increase in titer	121 (0–1325)	412.5 (85–1325)	69 (0–846)	**<0.001**
Post-/pre-immunization titer ratio	4.8 (0.8–38.9)	9.3 (2.9–38.9)	3.9 (0.8–16.5)	**<0.001**

*^a^Normal and impaired PS responses as assessed by the serotype-specific assay and using the American Academy of Asthma, Allergy and Immunology criteria (2)*.

*The data are quoted as the median (range) or the number (percentage)*.

*p-Values were obtained using a Mann–Whitney test for quantitative variables and a chi-square or Fisher’s exact test for qualitative variables*.

*Significant p-values are highlighted in bold font*.

#### Test Results

There was no significant difference between the good responders’ and poor responders’ median (range) sampling periods (respectively, 6.0 (4.0–7.8) and 6.4 (4.1–7.9) weeks after immunization, *p* = 0.465). The median (range) number of responsive serotypes was 3 (0–7) for the study population as a whole, 2 (0–4) for poor responders and 6 (5–7) for good responders (*p* < 0.001). In the SSAs, the response varied markedly from one serotype to another for both good responders and poor responders. An example of the complexity of comparing SSA and OVA responses due to SSA heterogeneity is illustrated for two patients with high OVA values in Figure S1 in Supplementary Material. Nevertheless, a linear regression of the sum of seven individual antibody responses and the overall antibody titers yielded acceptable Pearson correlation coefficients of *r* = 0.47 and *r* = 0.56 for pre-and post-immunization antibody titers, respectively (*p* < 0.001 for both).

**Figure 1:**
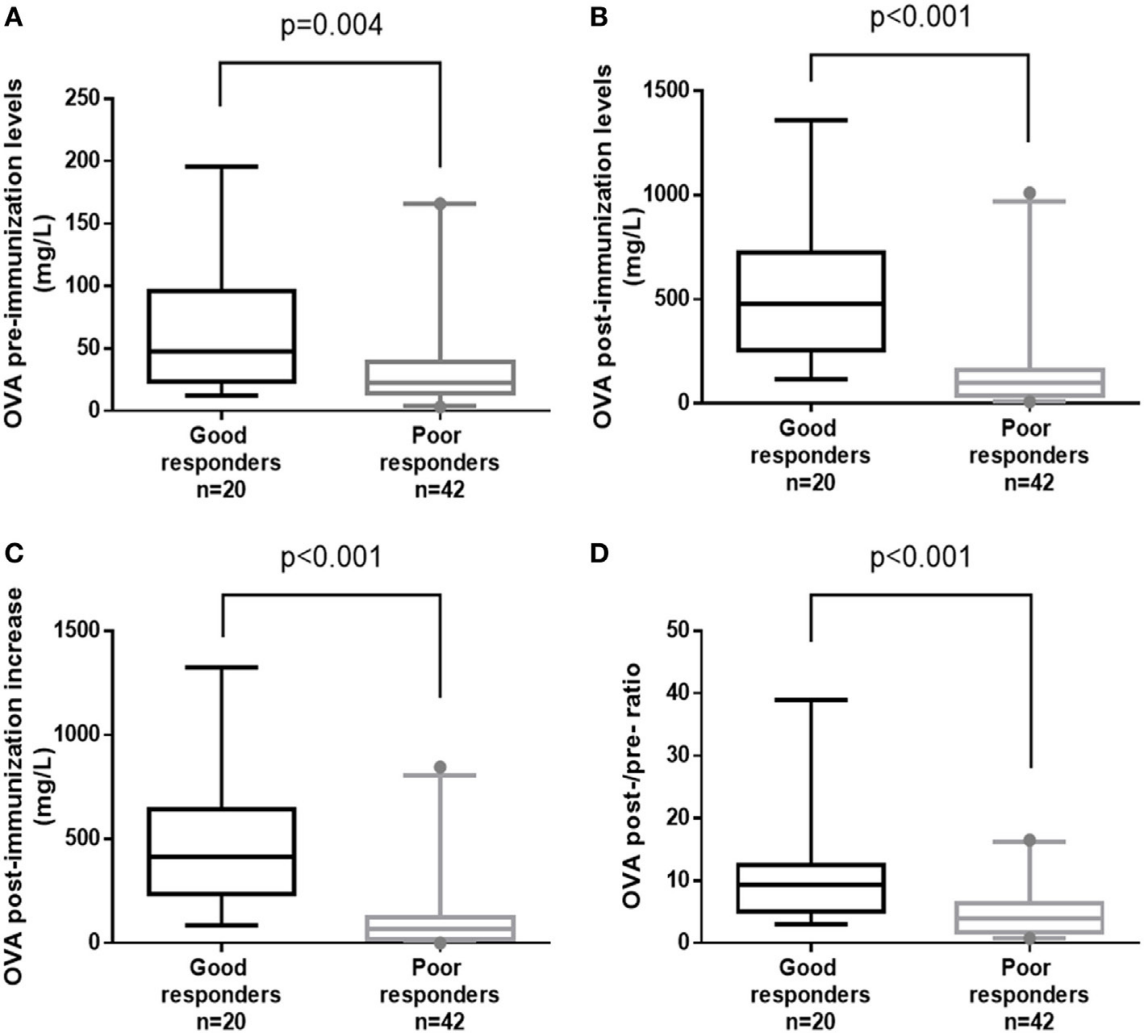
Box-plot representations of overall assay (OVA) antibody titers for good responders vs. poor responders, using the four indicated parameters: **(A)** OVA pre-immunization levels, **(B)** OVA post-immunization levels, **(C)** OVA post-immunization increase, and **(D)** OVA post-/pre-ratio. *p*-Values were calculated in a Mann–Whitney test. Boxes represent the median (50th percentile) and the 25th and 75th percentiles. Whiskers represent the 5th and 95th percentiles, and points are used to indicate extreme values.

A significant intergroup difference was observed for all the OVA criteria: the pre- and post-immunization titers (*p* = 0.004 and *p* < 0.001, respectively), the post-immunization increase and the post-/pre-immunization titer ratio (*p* < 0.001 for both) (Table 1; Figure 1). It is noteworthy that despite the presence of a significant difference in the median post-immunization titer between good responders and poor responders [480 (114–1360) and 100 (9–1010) mg/L, respectively; *p* < 0.001], several poor responders (*n* = 4, 9.5%) achieved high (>270 mg/L) post-immunization titers (Table 1; Figure 1).

**Figure 2:**
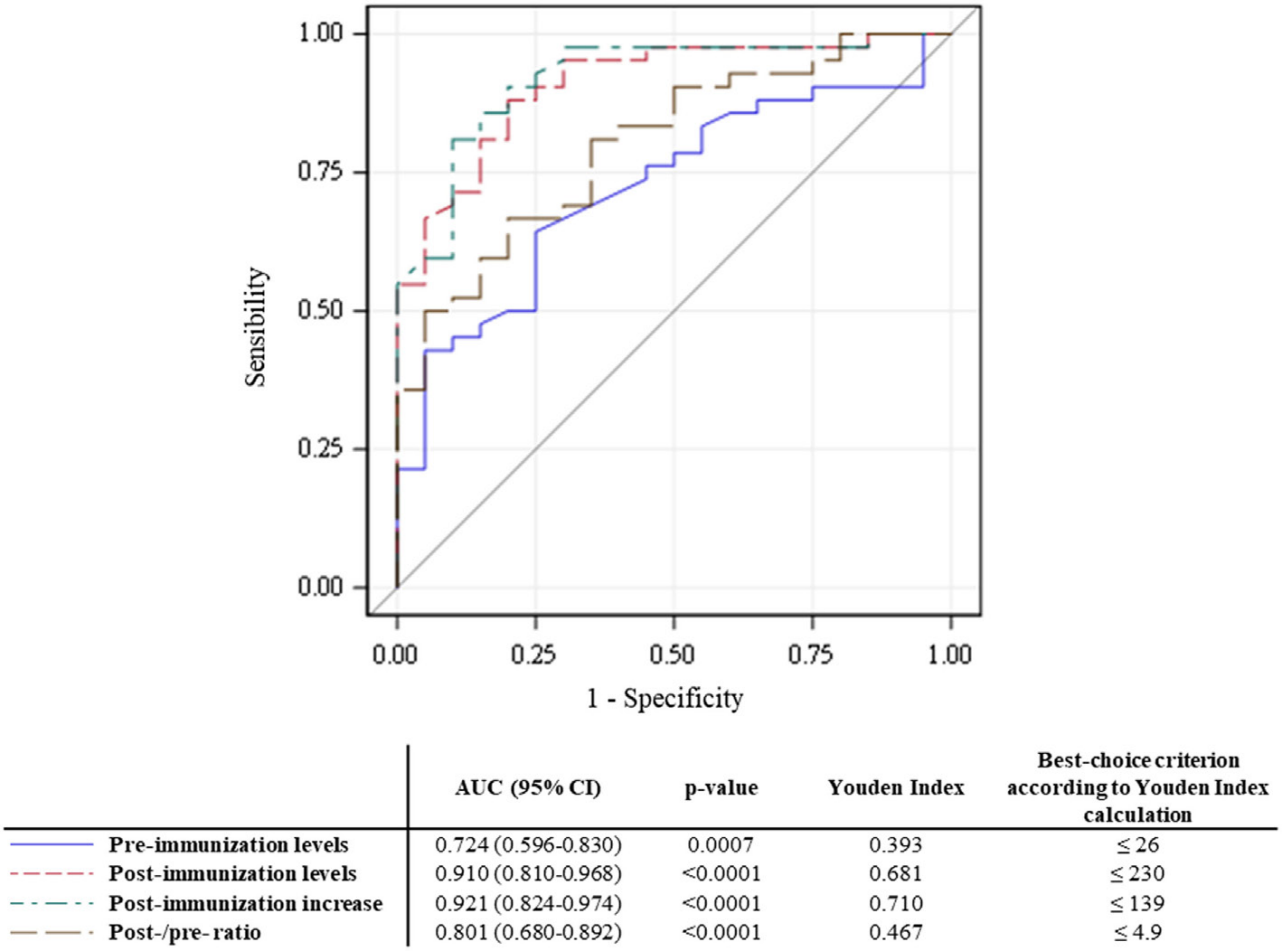
Receiver operating characteristic (ROC) curves for the four tested parameters. Estimated areas under the curve (AUCs) are presented with their (95% CI) and *p* values. The Youden index and best-choice criterion are indicated for each ROC curve.

The results of ROC curve analyses of the OVA’s performance vs. the SSA are summarized in Figure 2. In multiple AUC comparisons (data not shown), the post-immunization titer and the post-immunization increase discriminated between poor and good responders [AUCs, 95% confidence interval (CI) of 0.910 (95% CI, 0.810–0.968) and 0.921 (95% CI, 0.824–0.974), respectively] better than the pre-immunization titer and the post-/pre-ratio did.

Indicators of the OVA’s performance are presented in Table 2. In summary, an analysis of all four potential determinants showed that the Se increased and the Sp decreased as the magnitude of the response increased. Following calculation of the Youden index, the two best criteria were found to be a post-immunization antibody titer ≤230 mg/L and a post-immunization increase of ≤139 mg/L although neither of them achieved estimates of Se and Sp greater than 90%. As expected, the overall agreement was poor for most of the potentially determinant variables. McNemar’s test evidenced significant differences for a post-immunization titer of 230 mg/L (*p* = 0.008), and an increase of at least 139 mg/L (*p* = 0.049). Cohen’s kappa was never better than moderate (<0.60) for any of the criteria (Table 2).

**Table 2:** Performance indicators and levels of agreement for the overall assay (OVA)’s results.^a^

Criterion	Sensitivity (%)	Specificity (%)	Positive predictive value (%)	Negative predictive value (%)	McNemar’s test *p*-value	Cohen’s kappa
**Pre-immunization titer (mg/L)**						
≤26^b^	64.3 (48.0–78.4)	75.0 (50.9–91.3)	84.4 (67.2–94.7)	50.0 (31.3–68.7)	0.154	0,31 (0.08–0.53)
**Post-immunization titer (mg/L)**						
**≤110**	**57.8 (38.7–70.2)**	**100.0 (83.2–100.0)**	**100.0 (85.2–100.0)**	**51.3 (34.8–67.6)**	**0.761**	**0.53 (0.39–0.66)**
≤230^b^	88.1 (74.4–96.0)	80.0 (56.3–94.3)	90.2 (76.9–97.3)	76.2 (52.8–91.8)	0.008	0.52 (0.29–0.75)
**Post-immunization increase in titer (mg/L)**						
≤139^b^	81.0 (65.9–91.4)	90 (68.3–98.8)	94.4 (81.3–99.3)	69.2 (48.2–85.7)	0.049	0.57 (0.36–0.79)
**Post-/pre-ratio**						
≤4.9^b^	66.7 (50.5–80.4)	80 (56.3–94.3)	87.5 (71.0–96.5)	53.3 (34.3–71.7)	0.126	0.39 (0.17–0.60)

*^a^Comparatively to the vaccine response status as assessed by the serotype-specific assay and using the American Academy of Asthma, Allergy and Immunology criteria (2)*.

*^b^Selected using the Youden index. The selected threshold’s performances are highlighted in bold font*.

*All statistics are presented with the corresponding (95% confidence interval)*.

Given that our objective was to determine the value of first-intention use of the OVA, we searched for an easy-to-use criterion that maximized Sp (regardless of Se) than thus provided a reliable diagnosis. We found that a post-immunization threshold of 110 mg/L yielded the greatest Sp and correctly identified a high proportion of the poor responders [Se 57.8% (95% CI, 38.7–70.2); Sp 100.0% (95% CI, 83.2–100.0)].

When analyzing a broader set of 16 anti-serotype titers, only 1 of the 20 tested patients (5.0%) was reclassified (from a good responder to a poor responder). This 18-year-old woman presented with hypogammaglobulinemia and recurrent respiratory tract infections. She showed a good response for 5 of the first 7 serotypes tested in SSA but for only 11 of the 16 serotypes in total. In the OVA, the pre- and post-immunization titers were 17 and 182 mg/L, respectively. Consequently, only a slight decrease in Se was noted [Se 56.1% (95% CI, 42.1–70.1), Sp 100% (95% CI, 81.4–100)] when re-calculating the selected performance values for a threshold of 110 mg/L.

### The Prospective Validation Study

#### Participants

Twenty patients were included in the validation study [17 females (85%); median (range) age at inclusion: 45.5 (28–63) years].

#### Diagnostic Performance of the OVA

Using the OVA assay, seven patients presented post-vaccination titers below the 110 mg/L threshold; of these, two patients with very low post-vaccination OVA titers (48 and 76 mg/L) were first categorized as good responders by the SSA performed on seven serotypes. Consequently, the OVA-estimated performances were as follows: Se: 62.5% [24.5–91.5]; Sp 83.3% [51.6–97.9]. It is noteworthy that the two patients with very low post-vaccination OVA titers presented a good response for five of the first seven serotypes tested. As for the case described above, analysis of the 16 serotypes with an SSA for these two patients resulted in reclassification, with good responses to, respectively, eight serotypes (50.0%) and 7 serotypes (43.8%). After the re-assignment of both patients to the PID group (both SPAD), the OVA assay gave satisfactory values for diagnostic performance (Se: 70.0% [34.8–93.3]; Sp: 100.0% [69.2–100.0]; positive predictive value: 100.0% [65.3–100.0]). The flow chart for the prospective validation study is available in Figure S2 in Supplementary Material, along with the OVA’s diagnostic performance.

## Discussion

The present study is the first to have investigated the diagnostic value of a widely available OVA commercial kit in patients with a suspected PID by using the reference SSA to identify good responders and poor responders. Given that we wanted to establish whether use of the OVA alone could reliably identify a poor responder in routine clinical practice, we favored a high Sp. The most value parameter was a post-immunization titer below or equal to 110 mg/L [Sp 100% (95% CI, 83.2–100)]. However, the application of this cut-off value detected only around half of the poor responders [Se 57.8% (95% CI, 38.7–70.2)]—confirming that the SSA is required in a two-step procedure for patients with a post-immunization titer above 110 mg/L.

In a context of suspected PID, an anti-PnPS response assessment is of great clinical value. However, the gold-standard assay may prove both difficult to access and expensive to implement. The great inter-serotype heterogeneity of the anti-PnPS response in a given patient and the large inter-patient heterogeneity for a given serotype (as illustrated in Figure S1 in Supplementary Material) have long been recognized as important issues in assessment of anti-PnPS responses (9, 10). An isolated, elevated response to a single serotype may indeed be responsible for a high OVA value (18) (Figure S1 in Supplementary Material).

To overcome the heterogeneity problem, a number of multiplexed tests have been developed and evaluated for the diagnosis of impaired anti-PS responses. Many studies have evaluated the performance of Luminex^®^-based assays based on either in-house protocols or the 14-valent kit sold by Luminex Corporation (Austin, TX, USA) (6, 19), and several researchers have proposed specific cut-off values and/or qualitative criteria for the interpretation of Luminex^®^ results—thus enabling the use of this time- and cost-saving method (6, 7, 20–24).

Other types of assays have been proposed for the assessment of anti-PS immunization in suspected cases of PID. The results for measurement of allohemagglutinins did not support the use of this approach for assessment of the response to PSs (25). More recently, *Salmonella Typhi* Vi vaccination was investigated as an alternative tool for PS response assessment (26). Primary results were promising but patients and physicians might be reluctant to use the Typhim Vi vaccine systemically and the assay appeared to be less performant in case of previous immunization/infection. Although cut-off values for an *S. typhi* Vi vaccine response were recently proposed (7), pneumococcal vaccination is still the reference for assessment of the response to PSs (15). In this context, we sought to determine the diagnostic value of one of the assays most frequently used in routine practice.

Linear regression of the sum of seven individual SSA values yielded a low correlation coefficient for the pre-immunization titer (*r* = 0.47), and a slightly higher coefficient for the post-immunization titer (*r* = 0.56). In the subset of patients in whom the antibody responses to 16 serotypes had been measured, the pre-immunization titer again had a low correlation coefficient (*r* = 0.47). By contrast, the coefficient for the post-immunization titer was much higher (*r* = 0.92). Our present results are consistent with the literature data (13, 16). The increase in the correlation coefficient was expected, since anti-pneumococcal antibodies are known to bind more specifically to their antigens after immunization (27).

To assess the relevance of the OVA’s values, we tested four different parameters: the pre-immunization titer, the post-immunization titer, the total post-immunization increase, and the post-/pre-immunization ratio. On the population level, comparisons of the raw OVA data identified significant differences between good responders and poor responders for all four parameters (Table 1; Figure 1). In another study of the OVA, Sánchez-Ramón et al. reported significant differences in the pre- and post-immunization titers between CVID patients and healthy controls [*p* = 0.02 and *p* = 0.006, respectively (26)]. We expected the pre-immunization titer to discriminate less well between good responders and poor responders, since basal pneumococcal antibody titers mainly depend on previous infections and immunizations (2). On the individual level, our ROC curve analyses revealed significantly greater AUCs for the post-immunization titer and the post-immunization increase. Although we investigated the best values according to the Youden index, a satisfactory threshold was not found. Given that several criteria yielded non-significant differences in McNemar’s test, Cohen’s agreement statistic was consistently low—ruling out the reliable use of the OVA alone.

Since our aim was to integrate the OVA into a combined diagnosis approach (rather than replace the SSA), we nevertheless investigated the relevance of a two-step process in which the OVA assay could be used as a screening test for impaired PS responses. Intuitively (and despite the problem of response heterogeneity), patients who fail to increase their antibody titers and/or have rather low overall titers could possibly be bad responders. Accordingly, we determined that a post-immunization titer ≤110 mg/L had diagnostic value as the highest OVA value that would always identify poor responders [accounting for around half of the positive cases: Se 57.8% (95% CI, 38.7–70.2), Sp 100% (95% CI, 83.2–100.0)].

To validate our results, we measured nine additional anti-serotype antibody titers in 20 patients for whom sufficient volumes of serum had been collected before and after administration of PPV23. Only one patient (5.0%) was reclassified as a poor responder rather than a good responder, despite a relatively similar proportion of good serotype responses [from *n* = 5 out of 7 (71.4%) to *n* = 11 out of 16 (68.8%)]. These data emphasize that a seven-SSA analysis is reliable. After recalculation, the Se and Sp for a ≤110 mg/L threshold were, respectively, 56.1% (95% CI, 42.1–70.1) and 100% (95% CI, 81.4–100).

Lastly, we evaluated the threshold of 110 mg/L on 20 additional patients. Given that reclassification (from good responders to bad responders) had been required for the 16-serotypes SSA, we checked the two patients with very low post-immunization OVA levels and who had initially been classified as good responders. Using the 16-serotypes SSA, both were reclassified as poor responders, and so the OVA’s diagnostic performance with the 110 mg/L threshold was similar to that observed in the retrospective study [Se: 70.0% (34.8–93.3); Sp: 100.0% (69.2–100.0); positive predictive value: 100.0% (65.3–100.0)] (Figure S2 in Supplementary Material).

Our study has several limitations. The study participants were all adults and had been referred for a suspected PID. Only 42 (67.7%) received a final diagnosis of PID—thanks to an analysis of their PPV23 response, in many cases. It has been reported that the likelihood of identifying a PID is surprisingly high when adult patients are referred to specialist centers (28). This high diagnosis rate may also be related to our selection procedure, which was based on referral for SSAs: in our establishments, only highly probable cases are tested with SSAs in order to obtain a diagnosis and confirm an indication for immunoglobulin replacement therapy. Nevertheless, we also included some good responders, and we used the most recent, widely accepted interpretation criteria to evaluate our results.

Concerns about the specificity of the gold-standard SSA method were recently raised, since one study found that about 11.2 to 20.4% of healthy adults (depending on the criteria applied) with no history of infection had PS antibody deficiencies (29). Given that the latter study was based on a bead-based serotype-specific assay, it may suffer from a lack of specificity. This was suggested in previous studies in which the absolute antibody concentrations obtained by both methods remained different, despite a good overall correlation (11, 21, 30, 31). In a recent study, the authors advanced that AAAAI criteria could not be applied to SSA and to an in-house Luminex^®^ bead-based assay (7). However, the use of a 3-serotypes SSA and a shorter (3–4 weeks) time interval between immunization and sample collection could also explain some weak responses and that 11 to 34% of the healthy subjects were classified as SPAD with the SSA or with the bead-based assay, respectively. To conclude, the authors suggested some interesting fifth percentile cut-off values for the interpretation of the PPV response measured by their in-house bead-based assay, as a way to overcome this possible lack of specificity (7).

Despite the clear need for further studies of the clinical relevance of an impaired antibody response to PS in subjects without any medical history suggestive of PID, and considering that all our patients had recurrent and/or severe unexplained bacterial infections consistent with a possible antibody deficiency, we believe that this potential source of bias was limited in the present study (7, 15, 29).

It should also be noted that our approach cannot distinguish between the four SPAD phenotypes (namely mild, moderate, severe, and memory) (2, 32). Nevertheless, the SPAD phenotype has never been linked to clinical presentations and/or complications—although very recent data suggest that it might be of value in guiding treatment decisions (15).

**Figure 3:**
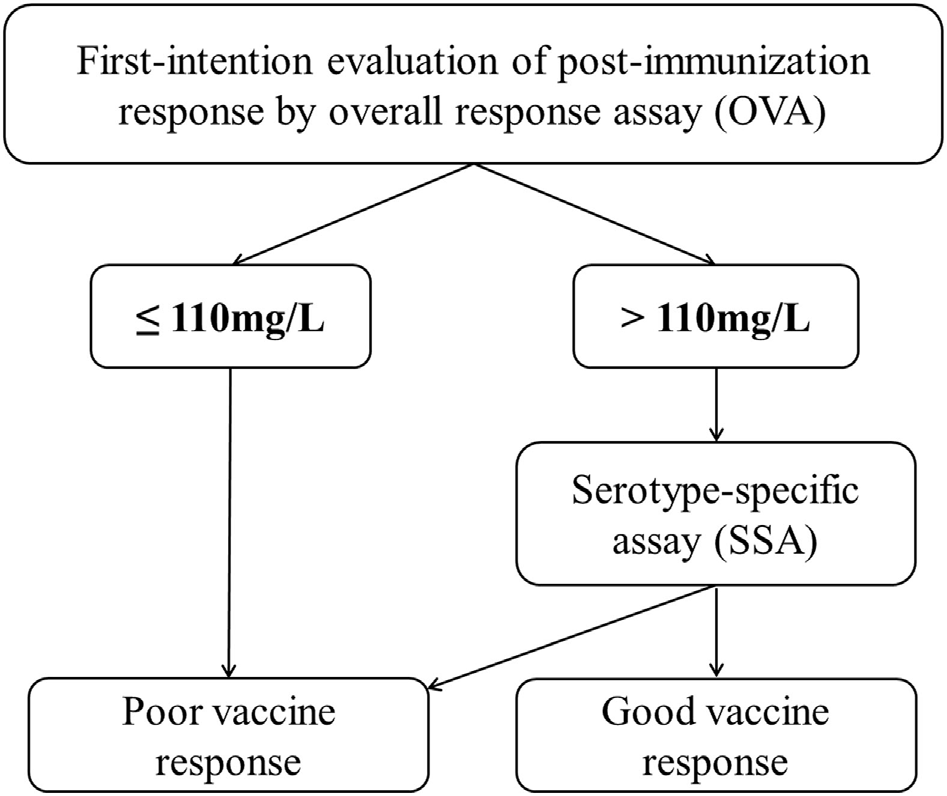
A decision tree using the overall assay (OVA) as a first-line test and SSAs for definitive assessment of the anti-PnPS response.

No patient with a post-immunization overall antibody titer ≤110 mg/L exhibited a good response using the 16-serotypes SSA. Indeed, a post-immunization overall antibody titer ≤110 mg/L yielded maximum specificity [100% (95% CI, 83.2–100.0)] and a high positive predictive value [100% (95% CI, 85.2–100.0)], although the high prevalence of poor responders in our population means that these predictive values should be confirmed in a larger cohort. Consequently, an impaired response can be diagnosed if the post-immunization overall antibody titer is ≤110 mg/L. However, the observation of higher antibody titers would require the application of SSAs for a definitive diagnosis. Accordingly, we propose a two-step diagnostic approach based on OVA screening and the use of SSAs when the post-immunization titer is >110 mg/L (Figure 3).

Finally, we evaluated only one commercial overall anti-pneumococcal response assay, and a similar comparison method should be applied to other commercial and in-house overall assays.

## Conclusion

We evaluated the diagnostic performance of an OVA for the assessment of impaired anti-PS responses. If the gold standard, WHO-validated SSA is not available, a commercial OVA may be of value in screening for PIDs. Our results suggest that a post-vaccination antibody titer below 110 mg/L in the OVA is associated with a poor PPV23 response. In all other cases, SSA is the only reliable method for assessing the response to PPV23.

## Ethics Statement

Concerning the patients (*n* = 33/82) included through the above-mentioned ongoing adult PIDs screening (ClinicalTrials ref: NCT02972281), this study was carried out in accordance with the recommendations of the Helsinki Declaration and complied with the requirements of the French Commission Nationale Informatique et Libertés, with written informed consent from all subjects. The reference code of the Agence Nationale de Sécurité du Médicament et des produits de santé (ANSM) is 140857B-61. Concerning the other patients (*n* = 49/82), the used samples came from a collection declared to the French Ministry in charge of Research (No. DC-2008-642). In accordance with French regulations, the process for declaring human biological collections for research purposes involves an independent ethics committee (i.e., the committee for the protection of individuals) and the opinion of this committee is decision-making. Consequently, it is recognized that the authorization issued by the Ministry in charge of Research takes the place of advice from the ethics committee.

## Author Contributions

BL and GL designed the study, analyzed and interpreted the data, and drafted the manuscript. CF, LT, DL, and GL have contributed to patients’ recruitment and data acquisition, and provided critical revision of the manuscript. MB, SM, MJ, and SP were responsible for parts of the data acquisition and contributed to critically revise the manuscript. SR, SB, A-SD, SD, FB, and ML supplied help to data interpretation and revision of the manuscript. All authors gave final approval of the version to be published.

## Conflict of Interest Statement

VaccZyme™ Anti-PCP human IgG ELISA kits were donated by the Binding Site Group Ltd., Birmingham, UK. Binding Site Group Ltd. had no role in experimental design or analysis. BL received travel and accomodation support from the Binding Site Group Ltd. for a congress. There are no other financial or commercial relationships to declare.

## Supplementary Material

Figure S1 Examples of discrepancies between SSA and overall assay (OVA) results for two representative patients. Antibody titers are displayed as histograms (blue: pre-immunization, red: post-immunization). The dotted line corresponds to the 1.3 mg/L cutoff for a good individual serotype response.

Figure S2 Flow chart for the prospective validation study. The numbers of patients are given with the overall assay (OVA) performances for the previously determined 110 mg/L threshold. Sp, specificity; PPV, positive predictive value.

## Abbreviations

AAAAI, American academy of asthma, allergy and immunology; AUC, area under the curve; CVID, common variable immunodeficiency; ELISA, enzyme-linked immunosorbent assay; OVA, overall response assay; PID, primary immunodeficiency; PPV23, 23-valent pneumococcal polysaccharide vaccine; PnPS, pneumococcal capsular polysaccharide; ROC, receiver operating characteristic; Se, sensitivity; Sp, specificity; SPAD, selective anti-polysaccharide antibody deficiency; SSA, serotype-specific assay; WHO, World Health Organization.
