# Supplementary figures and images for: Value of the Overall Pneumococcal Polysaccharide Response in the Diagnosis of Primary Humoral Immunodeficiencies
Source: Front Immunol. 2017 Dec 20;8:1862. doi: 10.3389/fimmu.2017.01862 (PMC5742330; doi:10.3389/fimmu.2017.01862)

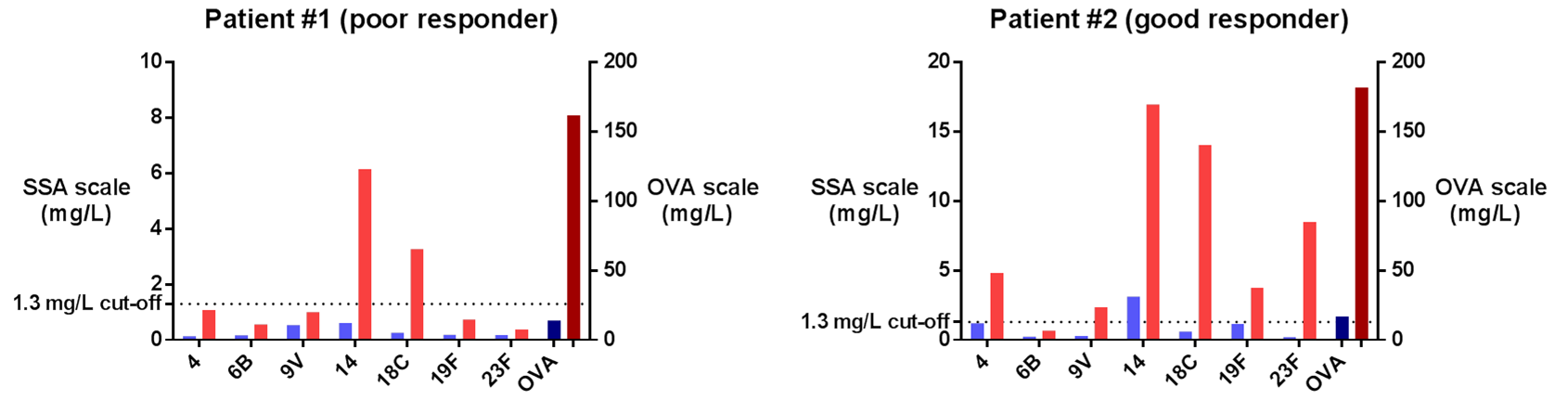

Supplement: Figure S1 — Examples of discrepancies between SSA and overall assay (OVA) results for two representative patients. Antibody titers are displayed as histograms (blue: pre-immunization, red: post-immunization). The dotted line corresponds to the 1.3 mg/L cutoff for a good individual serotype response. [file Image_1.tif]

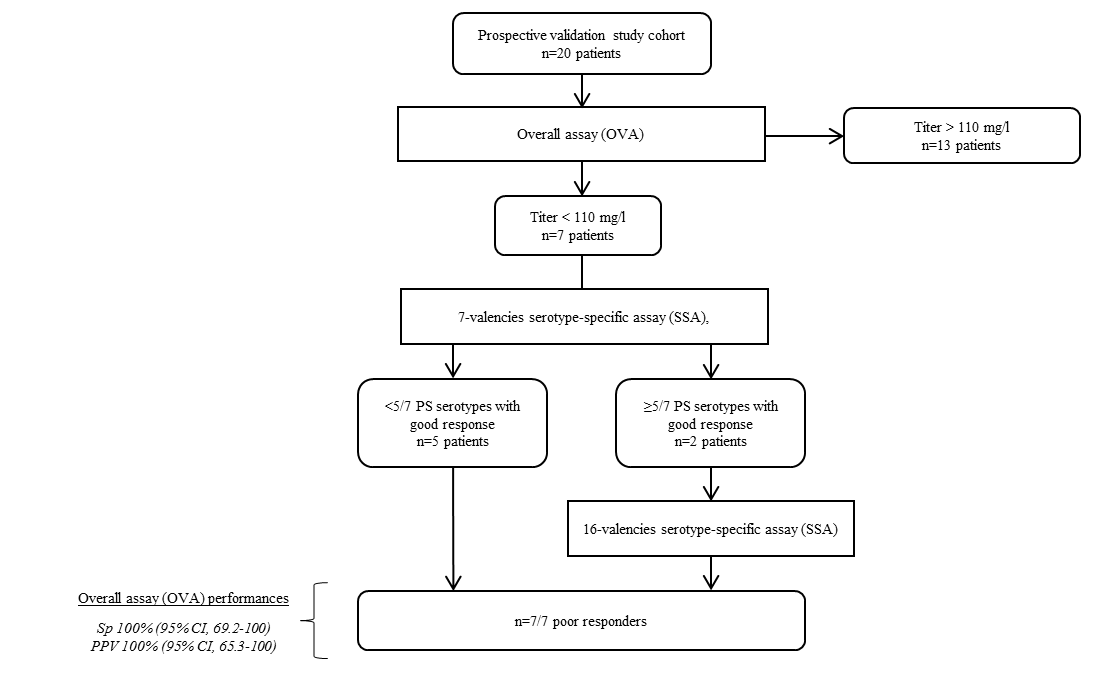

Supplement: Figure S2 — Flow chart for the prospective validation study. The numbers of patients are given with the overall assay (OVA) performances for the previously determined 110 mg/L threshold. Sp, specificity; PPV, positive predictive value. [file Image_2.tif]
